# Putting Psychology to the Test: Rethinking Model Evaluation Through Benchmarking and Prediction

**DOI:** 10.1177/25152459211026864

**Published:** 2021-09-23

**Authors:** Roberta Rocca, Tal Yarkoni

**Affiliations:** 1 Department of Psychology, University of Texas at Austin, Austin, Texas, USA; 2 Interacting Minds Centre, Aarhus University, Aarhus, Denmark

**Keywords:** psychology, model evaluation, benchmarking, machine learning, open data, open materials

## Abstract

Consensus on standards for evaluating models and theories is an integral part of every science. Nonetheless, in psychology, relatively little focus has been placed on defining reliable communal metrics to assess model performance. Evaluation practices are often idiosyncratic and are affected by a number of shortcomings (e.g., failure to assess models’ ability to generalize to unseen data) that make it difficult to discriminate between good and bad models. Drawing inspiration from fields such as machine learning and statistical genetics, we argue in favor of introducing common benchmarks as a means of overcoming the lack of reliable model evaluation criteria currently observed in psychology. We discuss a number of principles benchmarks should satisfy to achieve maximal utility, identify concrete steps the community could take to promote the development of such benchmarks, and address a number of potential pitfalls and concerns that may arise in the course of implementation. We argue that reaching consensus on common evaluation benchmarks will foster cumulative progress in psychology and encourage researchers to place heavier emphasis on the practical utility of scientific models.

The adherence to common standards for evaluating models and theories is an integral part of every science. A community of scientists that can find no agreement on what kind of work advances or hinders its goals is likely to experience difficulty making progress. A certain degree of flexibility in evaluation is, of course, both unavoidable and desirable: Researchers in every field regularly disagree with one another about the importance and implications of specific findings, and such differences of opinion are widely understood to be an important catalyst of theoretical and experimental advances. But even the most contentious scientific disagreements typically unfold against the backdrop of some shared understanding of what is and is not valid science: Astronomers do not tend to argue with astrologists in academic journals, nor do molecular biologists battle with creationists.

Psychology is, in many ways, a field with a healthy respect for consensus and convention. One need only peruse the pages of most psychology journals to observe a remarkable degree of convergence in method: Researchers tend to rely on similar patterns of reasoning, use similar statistical techniques, and even appeal to similar inferential quantities and binary thresholds (e.g., *p* < .05) to make critical decisions. Yet this convergence is, we argue in this article, deceptive in key respects: Beneath the superficial adherence to a common set of methodological conventions lies a realm of nearly unbounded evaluative freedom—one that imposes surprisingly few constraints on what researchers can or cannot do before they conclude that their preferred model is or is not supported by the available data.

Our argument is structured as follows. We begin by reviewing common evaluation practices in psychology and highlighting a number of features that have underappreciated but severe implications for our collective rate of progress. We then draw a contrast with evaluation practices in the field of machine learning, in which researchers place far greater emphasis on common benchmarks and out-of-sample, practically meaningful predictions. We argue that many areas of psychology would benefit considerably from the adoption of such practices and review a number of key desiderata for their successful implementation. We then discuss a number of examples within psychology in which consensus benchmarks are either already in use or have relatively clear paths to development and application. Finally, we consider common pitfalls and potential objections to the practices we have described. Our overarching conclusion is that convergence on consensus metrics and real-world benchmarks would advance the pursuit of traditional psychology goals (e.g., cumulatively building generalizable and reproducible knowledge) while simultaneously encouraging a focus on other highly desirable but historically underappreciated goals, such as maximizing the practical utility of scientific models and explanations.

## Model Evaluation in Psychology

Empirical sciences seek to develop theories and models that accurately describe and predict relations between empirical phenomena. In a seminal article, Breiman (2001) identified two main approaches to the problem of theory generation and validation. In the *data modeling* approach, researchers aim to develop models that reconstruct or approximate the true generative process underlying the data. In the *algorithmic modeling* approach, researchers proceed with few assumptions or ambitions with respect to the data-generating process; models are evaluated on their ability to produce desired outcomes (typically quantitative predictions) regardless of how they achieve that. The divide between data modeling and algorithmic modeling is paralleled by a distinction between *explanation-oriented* and *prediction-oriented* model evaluation (Shmueli, 2010). In the explanatory tradition, models are evaluated favorably if they are comprehensible to researchers and display desirable qualitative features (e.g., the ability to capture specific patterns of effects). In prediction-oriented approaches, models are typically evaluated according to their generalization performance at predictive tasks—that is, the ability to approximate or correctly label new observations drawn from the same or similar populations the models were trained on.

Traditionally, the overwhelming majority of scientific practice in psychology has fallen within the data modeling/explanatory tradition. Theories are typically developed into hypotheses consisting of qualitative predictions about the directionality or magnitude of specific effects—that is, hypotheses about how parameter estimates for predictors of interest will deviate from the values expected under a null hypothesis. These qualitative predictions are often corroborated or disconfirmed via quantitative procedures, and indicators of statistical significance are often complemented with metrics of model performance that quantify models’ ability to explain variance in the specific data sample.

The tendency to place heavier emphasis on qualitative effect patterns than on predictive accuracy is understandable in contexts in which, as it is often the case in psychology, one’s primary goal is to understand the internal structures that give rise to various aspects of mental function; merely demonstrating that a predictive model can quantitatively approximate specific behaviors provides no guarantee that it is capitalizing on similar processes as the human mind, let alone that its internal structure can be readily understood. Nevertheless, traditional model evaluation approaches in psychology suffer from a number of important weaknesses that threaten to undermine their ability to reliably assess how well a model is doing—even with respect to purely explanatory goals.

A first problem is that the ability of a model to display some desired qualitative pattern—for example, statistical significance or a good model fit—provides no guarantee that the model can actually do anything predictively useful. This is true even within the narrow context in which the effect was originally demonstrated: A model can produce highly significant effects of interest that nevertheless do not enable an analyst to nontrivially predict outcomes for new observations even when those observations are sampled from the same data-generating process as previously seen observations.

This problem is widely acknowledged in the literature, and it is often framed in terms of effect sizes—that is, that significance isn’t per se a guarantee of predictive validity; effect size also matters (Cumming, 2013; Kline, 2013; Nakagawa & Cuthill, 2007; Sullivan & Feinn, 2012). But effect-size-based evaluation approaches also have important limitations and can be contrasted with a predictive perspective that often reveals otherwise unavailable insights.

To illustrate, we reanalyzed data from the Many Labs Project (Klein et al., 2014)—a large-scale multisite replication project investigating the replicability of 13 classical psychology effects in large samples collected across multiple independent sites.^1^ The authors reported that 10 effects replicated consistently and had aggregate *p* values below .001. However, the magnitude of these 10 effects varied wildly (Cohen’s *d* range = 0.12–2.42). From a predictive standpoint, it is easy to demonstrate that even highly significant effects can produce essentially worthless predictions.

Consider two of the effects that putatively replicated consistently. First, take one of the anchoring effects (Jacowitz & Kahneman, 1995), in which participants are asked to provide an estimate of the height of Mount Everest after being exposed to unrealistically high or low anchor values. A simple least squares regression model predicting the outcome variable (the estimated height of Mount Everest) from only the experimental condition assignment explains 57% of the variance. However, this estimate is, in principle, susceptible to *overfitting* (for discussion, see Yarkoni & Westfall, 2017) because the same data are being used to fit the model and to evaluate its performance—in effect, double-dipping. What happens when we reanalyze the data using a cross-validation procedure that estimates the out-of-sample predictive performance using different subsets of the data to train and test the model? In this case, the answer is “not much.” The estimated out-of-sample *R*^2^ is still .57—implying that our trained regression model predicts outcomes for entirely new individuals drawn from the same distribution just as accurately as outcomes for individuals it has already seen.

**Fig. 1.**
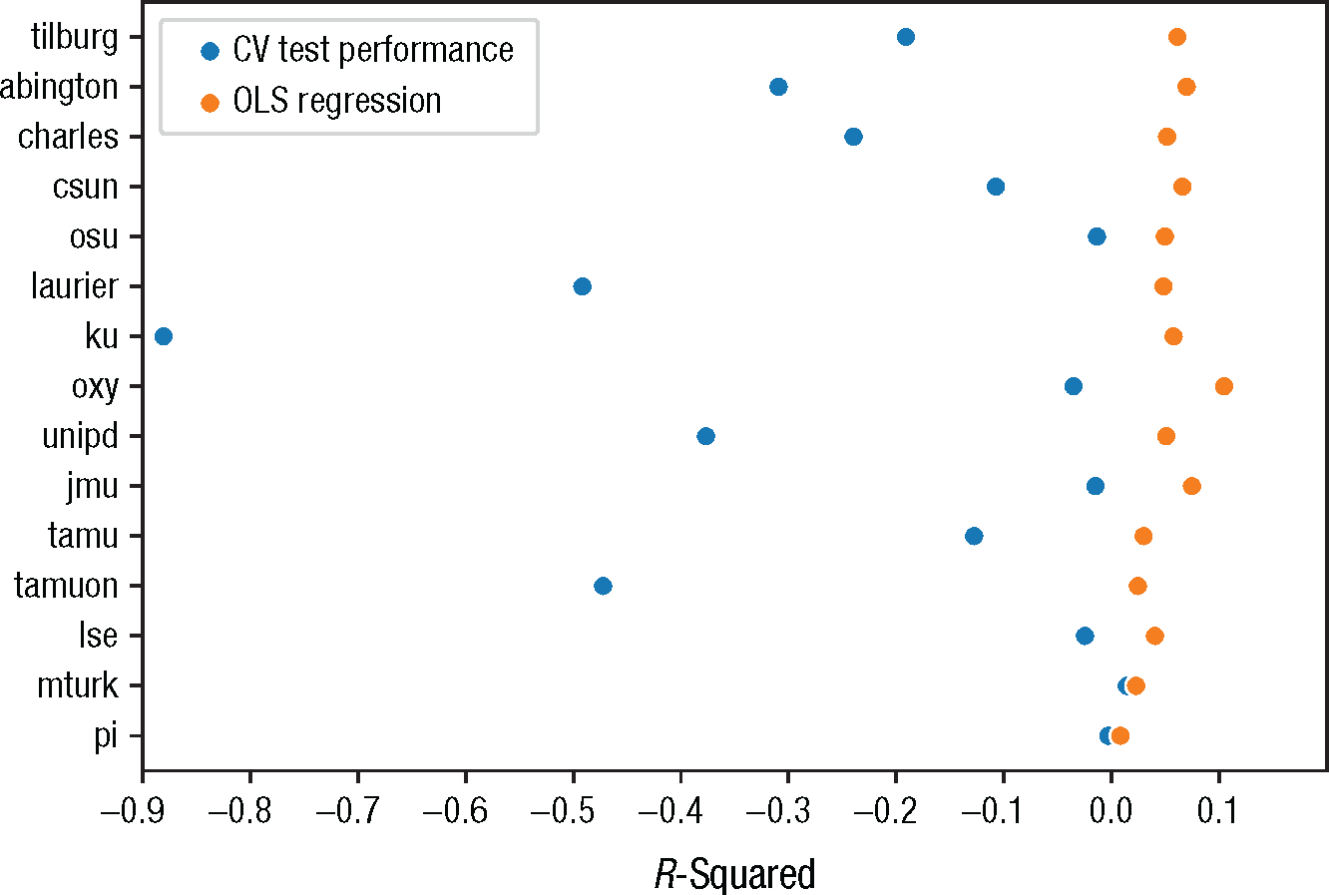
In-sample *R*^2^ and out-of-sample *R*^2^ (from 10-fold cross-validation) for sunk cost effects from Klein et al. (2014) for each experimental site in which the effect reaches statistical significance (*p* < .05). For many sites, in-sample and out-of-sample *R*^2^s differ widely because of overfitting. Inflated in-sample *R*^2^ can lead to overly optimistic interpretations of the true predictive capacity of a model when out-of-sample performance is disregarded.

By contrast, if we repeat the same analysis on the “sunk cost” effect in Many Labs—in which participants are asked to report how likely they would be to go see a football game on a freezing cold day assuming that they either paid for their ticket or were given it for free—we get a very different picture. Here, the in-sample *R*^2^ is an already modest .02, but the out-of-sample *R*^2^ is actually negative (−.02).^2^ In other words, it is not just that the effect is “statistically significant, but small,” as a traditional interpretation might suggest; the model is actually completely incapable of using knowledge of the experimental assignment to improve its prediction of game attendance in new individuals. Worse still, the above analysis is arguably something of a best-case scenario because the large training sample naturally mitigates overfitting (we return to this point later). If we fit separate models for each experimental site (i.e., for sample sizes varying between 79 and 1,323 participants), we observe a *p* value below .05 for 15 out of 36 sites. For all but one of these sites, the out-of-sample *R*^2^ is negative, but the in-sample *R*^2^ ranges up to .08 (for *n* = 174; see Fig. 1). In this scenario—which is arguably much closer to the modal psychology study in terms of sample size and reported *p* value—we might draw radically different conclusions about the importance or utility of our model if we failed to consider its true (i.e., out-of-sample) predictive capacity.

Given this finding, should one still count the “consistent replication” of the sunk cost effect reported in Many Labs (as expressed by *p* values) as support for any theoretical hypothesis? Maybe—but it is far from obvious. And similar problems will often go unnoticed when using conventional evaluation procedures. Researchers who focus exclusively on in-sample statistics might never realize that a statistical model they interpret as support for some psychological hypothesis may be incapable of generating any meaningful prediction even when applied to new data sampled from exactly the same distribution, let alone to data obtained from a different measurement context.

A second problem is that many of the procedures psychologists traditionally use to evaluate their theories and models are widely misunderstood, and consequently, misinterpretation of results is common. It has been observed for decades, for example, that most psychologists do not appear to understand the meaning of the *p* values they so avidly use as decision aids (Gelman, 2013; Greenland, 2011; McShane et al., 2019). The coefficient of determination (*R*^2^) is frequently interpreted as a general measure of a model’s predictive capacity, yet it does not, by itself, indicate whether a model is capturing relevant and generalizable patterns in the data or is merely overfitting to spurious error sources (e.g., measurement error; J. I. Myung & Pitt, 2018). A whole host of inferential mistakes routinely arise when researchers draw unsupported causal inferences from observational data using standard regression methods—for example, treating regression coefficients as causal estimates without disclosing and defending causal assumptions (Rohrer, 2018; Watts, 2014; Watts et al., 2018) or concluding that a construct of interest has incremental validity over and above other constructs simply because a given measure of that construct makes a statistically significant contribution after “controlling for” putative confounds (Kahneman, 1965; Shear & Zumbo, 2013; Westfall & Yarkoni, 2016).

A third weakness of standard evaluation practices is their marked idiosyncrasy. The choice of empirical and statistical operationalization of a given hypothesis and the concrete implementation of the analysis workflow are typically left almost entirely to the discretion of researchers’ individual preferences. And researchers freely exercise that privilege. If theories in psychology are like toothbrushes, in that “everyone has one, and nobody wants to use anyone else’s” (Mischel, 2008), operationalizations of theories are, perhaps, like pieces of chewing gum: Many researchers do not seem to want to use even their own a second time.

We are not suggesting that this norm is unjustified; there are often excellent reasons why a particular theory or model cannot be rigorously tested without introducing new experimental designs or methodological procedures to the literature. But the practice clearly also has heavy costs. Chief among these is a lack of standardization, which severely limits the commensurability of different studies—even those nominally seeking to test competing theories of the same phenomenon. When two psychologists disagree about the merits of their respective theories, it can be difficult to determine where the disagreement actually resides. Are there competing interpretations of the same data point? Is the disagreement over whether a given experiment is a good test of a theory? Do the theories actually differ in scope despite using similar nomenclature?

Absent consensus benchmarks that encourage commensurability and provide at least a partial basis for objective evaluation of different researchers’ output, there is little to prevent people from adopting whichever procedures and emphasizing whichever results they find most expedient. It can be difficult for a field to make collective progress when individual researchers get to decide for themselves, using their own idiosyncratic evaluation criteria, how well they are doing. The fact that researchers overwhelmingly justify their evaluations using similar quantitative criteria is of little help: It does one little good to know that 90% of the researchers in a field agree that a *p* value below .05 means one’s hypothesis is corroborated so long as each researcher gets to freely define the hypotheses, measurement context, and statistical model that produce that *p* value.

In highlighting these weaknesses, we are not trying to add fuel to the long-burning fire of statistical criticism aimed at psychologists. What is salient to the present argument is not so much the observation that psychologists frequently engage in suboptimal evaluation practices, it is that extended contact with empirical data rarely seems to provide much corrective feedback. What does it imply about the reliability of a discipline’s output if a large fraction of its practitioners can spend their careers “successfully” using standard evaluation procedures without reality ever intruding onto and correcting fundamental misconceptions about those same procedures? When civil engineers screw up their calculations, bridges fall down; when physicists make basic mathematical errors, those mistakes ramify quickly to the point of detection and correction; even new drivers who fail to maintain sufficient distance from the cars in front of them is likely to learn some caution the hard way. Yet in most areas of psychology, a researcher can spend years happily using *p* values to decide whether theories should live or die and still come away confidently asserting that a *p* value below .05 means there is a 95% probability the hypothesis is correct. If standard evaluation procedures in psychology cannot be trusted even to identify and rectify glaring misunderstandings about those procedures themselves, how can they be trusted to assess the quality of psychological theories?

These fundamental issues highlight the need to rethink evaluation practices in favor of more reliable consensus procedures in which models are regularly submitted to solid “reality checks.” To achieve this, researchers may be better off drawing inspiration from prediction-focused fields like machine learning, in which there is a strong emphasis on evaluating model performance using consensus data sets, out-of-sample prediction metrics, and benchmarks that have real-world implications. In the next sections, we review a number of these practices and discuss ways psychology can learn from some of the strengths of the machine-learning model. In urging psychologists to draw inspiration from machine learning, we do not intend to ignore the potential drawbacks of exclusive focus on predictive accuracy (we expand on these in later sections), nor do we intend to encourage the field to relinquish its ambition to build cumulative knowledge on empirical phenomena. As we discuss in detail later, prediction and knowledge building are not only compatible but also deeply intertwined. At the affordable cost of rethinking explanation in terms other than direct interpretation of estimates from oversimplified models (i.e., with the aid, for example, of interpretability techniques), psychology has a lot to gain from substantiating its theoretical claims with evidence of their real-world predictive value in terms of reliability, validity, and potential for incremental progress.

## Model Evaluation in Machine Learning

*Benchmarking* refers to the practice of evaluating models by testing their predictive performance in tasks that reliably represent the purpose they are developed for (e.g., a scenario that mimics a plausible deployment context). A benchmark thus consists of two core components: a data set in which each example is coded along relevant input features and target measures and a clear task specification defining which metrics should be used to quantify the model’s predictive accuracy. Widespread consensus on reference benchmarks is crucial in fields that rely on benchmarking as their main evaluation protocol. Adoption of standard evaluation data sets, tasks, and metrics enables the community to compare models on objective grounds and to set realistic expectations on the predictive accuracy for future work.

The practice of evaluating scientific models on public benchmarks is well entrenched in machine-learning research. The natively applied nature of much machine-learning research is, arguably, one of the main reasons for this: Machine-learning models are often explicitly designed with concrete deployment scenarios in mind in which achieving high accuracy on individual predictions is the model’s primary goal. Relatedly, widespread use of benchmarks has followed naturally from the field’s long track record in building and sharing large data sets. Developing data sets of size suitable to efficiently train machine-learning models—and particularly highly parameterized deep neural networks—has historically been resource intensive and time-consuming, which has encouraged researchers to coordinate data-collection efforts and publicly share the resulting resources. As a consequence, the presence of consensus data sets has naturally provided to the machine-learning community a set of reference empirical problems on which models can be evaluated and compared.

**Fig. 2.**
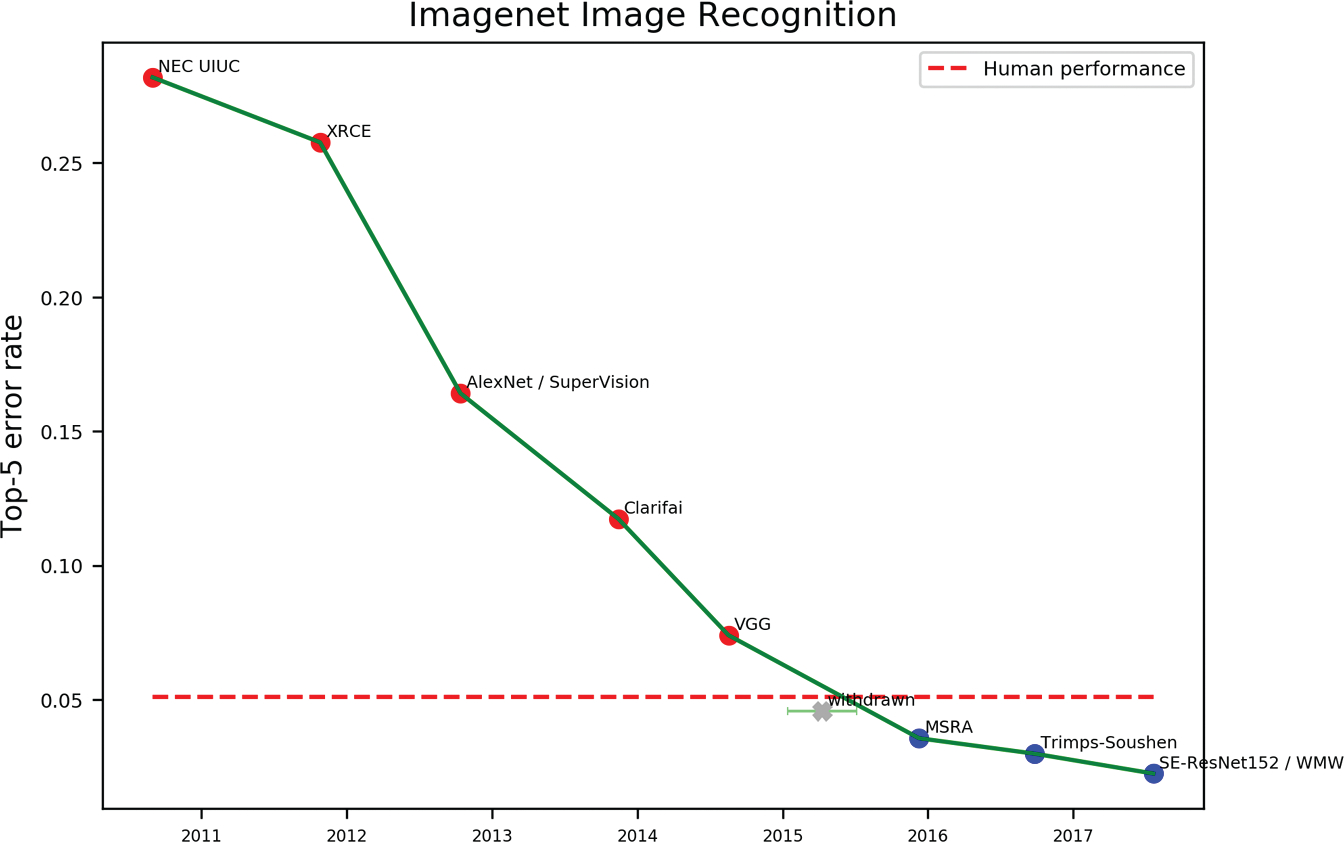
Performance of computer-vision models on the ImageNet challenge over time (2010–2017). The release of ImageNet has strongly incentivized the development of increasingly more powerful computer-vision architectures. AlexNet was a crucial breakthrough, achieving a 10% drop in error rate compared with the previous models. Current state-of-the-art models outperform humans. Retrieved from https://www.eff.org/ai/metrics.

Some milestone data sets have proven especially central to the development of the field, directly triggering the impressive advances observed, for example, in computer vision and natural language processing over the last decades. The most popular and perhaps most influential example is ImageNet (Deng et al., 2009), a massive, continuously updated database of labeled images of objects built on the WordNet ontology (Miller et al., 1990). At present, ImageNet includes around 14 million images displaying objects belonging to more than 20,000 object categories. ImageNet lends itself to evaluating model performance on a number of computer-vision tasks, such as object detection (identifying the presence of an object in an image), image classification (correctly classifying the type of object), and image segmentation (segmenting the image into distinct objects), and it is still one of the most widely used benchmarks in the field. Crucially, the release of ImageNet was followed by the launch of the annual ImageNet Large Scale Visual Recognition Challenge (ILSVRC), in which machine-learning researchers competed to develop models able to perform well on a 1,000-class image-classification problem. It was in the attempt to solve the ImageNet challenge that the AlexNet model (Krizhevsky et al., 2012)—often pinpointed as a critical milestone in the “deep learning revolution” (LeCun et al., 2015)—emerged (Fig. 2).

The ImageNet case is a textbook example of how cleverly designed benchmarks posing challenging predictive problems can boost progress in research targeting key long-standing problems. But the introduction of new benchmarks can also contribute to shifting the focus toward less developed research topics. One example of this is the rising interest in the problem of grounded language learning within natural language processing. Most traditional language models are trained exclusively on text: Models learn high-dimensional linguistic representations from statistical patterns in massive corpora (e.g., CommonCrawl), a strategy that has proven effective for many applications of artificial language systems. State-of-the-art transformer models (Devlin et al., 2018; Vaswani et al., 2017) achieve previously unthinkable levels of performance even on challenging tasks such as question answering and summarization. However, many instances of real-world language rely on joint processing of linguistic input and sensory and motor information (e.g., spatial navigation requires linking words to visual input and actions), and most current language models lack direct access to nonlinguistic inputs. The need to integrate sensorimotor knowledge into the semantic representations learned by language models has recently led to the development of a number of benchmarks tackling vision-and-language tasks aimed at encouraging the community to focus on this challenging subdomain (Chen et al., 2020; Shridhar et al., 2020). Notable examples are Touchdown (https://sites.google.com/view/streetlearn/touchdown), which evaluates models’ ability to locate objects in a real-life visual environment using a combination of linguistic instructions and either egocentric or allocentric spatial descriptions, and Alfred (https://askforalfred.com/), which probes models’ ability to correctly perform a range of actions using natural language task descriptions, instructions, and visual demonstrations.

Another important consequence of the strong emphasis placed on benchmarks is that over time, the repertoire of available benchmarks has expanded and diversified, which makes it possible, in many domains, to test model performance on a battery of tasks and quickly gain a comprehensive overview of its strengths and liabilities. Once again, natural language processing offers an excellent example: Language models are generally trained on one or more general-purpose objectives (e.g., next-word prediction), and after (often minimal) fine-tuning, they are evaluated against composite benchmarks (e.g., Sakaguchi et al., 2019; Wang et al., 2019). In this regard, a particularly interesting example is that of GPT-3 (T. B. Brown et al., 2020), a language model optimized for next-word prediction but whose architecture is entirely task agnostic. The model can thus be directly (i.e., without fine-tuning or gradient updates) evaluated on virtually any task that can be framed in terms of context-completion pairs using a learning framework, named *few-shot learning*, which mimics humans’ learning by demonstration (T. B. Brown et al., 2020). Using this framework, GPT-3 was evaluated on a wide range of well-established benchmark tasks, including next-word prediction (language modeling) on the Penn TreeBank data set (Marcus et al., 1994), long-range dependency modeling on the LAMBADA data set (Paperno et al., 2016), story completion on HellaSwag (Zellers et al., 2019) and StoryCloze (Mostafazadeh et al., 2016), and Winograd pronoun resolution tasks (Lin et al., 2020; Sakaguchi et al., 2020), as well as novel ones, capturing, for example, the model’s ability to learn and use new words or to perform symbolic manipulations of words (e.g., reversing or reconstructing scrambled words). This simple but groundbreaking evaluation protocol has enabled researchers to quickly gain a comprehensive overview of GPT-3’s strengths (e.g., in tasks such as modeling of long-range dependencies) and weaknesses (especially apparent in tasks such as commonsense reasoning or arithmetic tasks), paving the way toward further improvements.

Finally, emphasis on benchmarks has also ensured steady methodological improvements in a number of domains. One notable example is the development of standardized protocols for minimizing overfitting, which are now gaining popularity in many other fields. The theory and practice of cross-validation and regularization methods (e.g., lasso and ridge regression for linear models or dropout for neural networks) was largely developed in statistics and machine learning (although it is intriguing to note that some of these methods have their initial roots in psychometrics; e.g., Mosier, 1951) alongside practices that aim to minimize overfitting during model selection and pipeline construction (e.g., the custom of holding out test data sets in predictive modeling competitions).

## Toward Useful Metrics and Benchmarks

Machine learning’s pervasive reliance on objective performance benchmarks arguably follows quite naturally from its practitioners’ tendency toward an algorithmic-modeling philosophy. The situation in psychology is somewhat different. There have certainly been efforts to standardize evaluation practices, ranging from explicit attempts at building benchmarks for working memory research (Oberauer et al., 2018) to meta-analyses of effect sizes (Bosco et al., 2015; Hemphill, 2003; Hill et al., 2008; Paterson et al., 2016; Plonsky & Oswald, 2014; Richard et al., 2003; Taylor et al., 2018; van Erp et al., 2017; Vanhove & Harms, 2015) aimed at informing study design and setting reasonable priors for study outcomes. However, these valuable contributions still overwhelmingly rely on in-sample estimates rather than out-of-sample prediction of individual data points. Furthermore, they are the exceptions in a field that has shown little overall inclination toward adoption of common benchmarks, and active (individual as well as community wide) efforts will be needed to successfully introduce many of the beneficial practices and conventions currently found in the machine-learning community into psychology. In this section, we discuss a number of core principles that would maximize the utility of benchmarks in psychology and identify concrete steps researchers can take to help realize them. We hasten to emphasize that these principles are neither necessary nor sufficient for success and are likely to vary widely in importance across different areas of research.

### Large data sets

As the case of machine learning shows, identifying or developing suitable data sets is often the first and most important step in developing good benchmarks. The most important criteria for a good benchmarking data set are that it includes large and highly variable samples on key dimensions of interest (e.g., participants, experimental paradigms, and stimulus sets; see Oberauer et al., 2018) and that it is publicly available and easily accessible. Two other characteristics are also desirable in many cases: Data should be collected in settings reflecting naturalistic conditions, which increases the ecological validity of high-performing models, and data sets should lend themselves to predictive tasks with explicit practical utility. Needless to say, standard considerations regarding limitations on data sharing apply here: Not all data sets that could serve as a good benchmark in principle can be used as such in practice.

In contrast to machine learning, psychology does not have a strong tradition of collaborative building and public sharing of large data sets (although we discuss several exceptions later). The advent of the big data era has been welcomed by many psychologists as a potential game changer, but its influence on research practices in most areas has thus far been minimal (Yarkoni & Westfall, 2017). To a large extent, psychologists continue to prioritize carefully tailored, small-sample, in-house experimental manipulations over large-scale analyses of general-purpose data sets produced by other researchers. Although there is much to be said for the ability to design experimental procedures that directly address one’s chosen research question, many psychologists fail to fully appreciate the countervailing costs—such as the degree to which small samples inflate effect sizes (Gelman & Weakliem, 2009; Ioannidis, 2008; Yarkoni, 2009) and how, in the presence of selection bias, false-positive rates (Button et al., 2013) are susceptible to statistical and procedural overfitting (Yarkoni & Westfall, 2017) and contribute to publication bias (Ferguson & Heene, 2012; Open Science Collaboration, 2015; Vasishth et al., 2018) and widespread generalization failures (Yarkoni, 2019).

Note that the acquisition of large data sets does not have to impose a huge burden on individual researchers. An effective strategy is to coordinate data collection across research groups and publicly share the resulting data sets. Large-scale efforts of this kind have already been conducted in psychology and neuroscience. Notable examples are the Psychological Science Accelerator (https://psysciacc.org/; see Moshontz et al., 2018), the ManyLabs (Klein et al., 2014) and ManyBabies (Frank et al., 2017) replication projects, the Human Connectome Project (Van Essen et al., 2013), BioBank (Allen et al., 2014), lexical data sets such as the English Lexicon Project (Balota et al., 2007) and the Small World of Words data set (De Deyne et al., 2016, 2019), large neuroimaging data sets such as the Naturalistic Neuroimaging Database (Aliko et al., 2020), the Narratives database (Nastase et al., 2019) and data sets from the Courtois project on neuronal modeling (CNeuroMod, https://www.cneuro mod.ca/), or the neuroimaging and genomics data set ENIGMA (http://enigma.ini.usc.edu/). Some of these data sets rely on gathering smaller amounts of data from large participant samples, whereas others sample more extensively from smaller participant pools. What the optimal strategy is depends largely on the problem at stake. In some cases, establishing shared “participant panels” allowing researchers to iteratively expand data sets with new sets of measures and tasks would be just as beneficial as data set building and sharing per se. Social science data sets such as the General Social Survey (https://gss.norc.org/), the World Values Survey (http://www.worldvaluessurvey.org), the World Inequality Database (https://wid.world/), and the American National Election Studies (https://electionstudies.org/) are also relevant to many areas of psychology research. Moreover, online data-collection platforms like Amazon Turk (https://www.mturk.com/) or Prolific (https://www.prolific.co/) and relatively novel data types such as social media data and mobile device sensors data often make development of large data sets even less effortful and resource intensive. Conditions are thus favorable to initiate and promote the creation of consortia that would harness these resources on a large scale.

The above-mentioned data sets are only a small sample of the resources publicly available today. Attempts to compile comprehensive lists of available data are currently being undertaken (see, e.g., https://osf.io/mbvk4/). These resources are an excellent starting point for researchers to gain an overview of the state of the art and identify data sets that can easily be expanded or simply repurposed as predictive benchmarks. Their use as benchmarks can be promoted via data articles that describe their characteristics and provide baseline models (Chavan & Penev, 2011; Gorgolewski et al., 2013).

### Good target measures and performance metrics

Development of good benchmarks relies on the choice of good target measures and performance metrics that adequately reflect a model’s predictive validity. To avoid some of the main downsides of commonly used metrics and ensure reliable evaluation, benchmarks should thus meet at least two important criteria.

First, target variables included in benchmark data sets should take seriously traditional psychometric considerations such as measurement reliability and construct validity. That is, a good target measure should primarily measure the phenomenon it is intended to measure and have minimal contribution from other constructs or methodological confounds. So stated, this point may seem obvious, but the history of psychology suggests it is far from trivial. In many domains of psychology, core constructs are routinely operationalized in highly idiosyncratic ways (Eisenberg et al., 2019), and logical fallacies in the operationalization of constructs are pervasive (Cronbach & Meehl, 1955; Meehl, 1978, 1990). Outside of a few subfields (e.g., personality and individual differences, psychopathology, and industrial-organizational [I/O] psychology), reliability and validity are rarely systematically assessed. Although good psychometric properties are desirable in any study, they are particularly critical when developing performance benchmarks likely to be used by other researchers because any biases or errors risk being widely (and usually silently) propagated. Identifying good evaluation metrics will thus require research communities to engage in constructive debate informed by thorough (logical and empirical) assessments of the validity of each candidate measure.

**Fig. 3.**
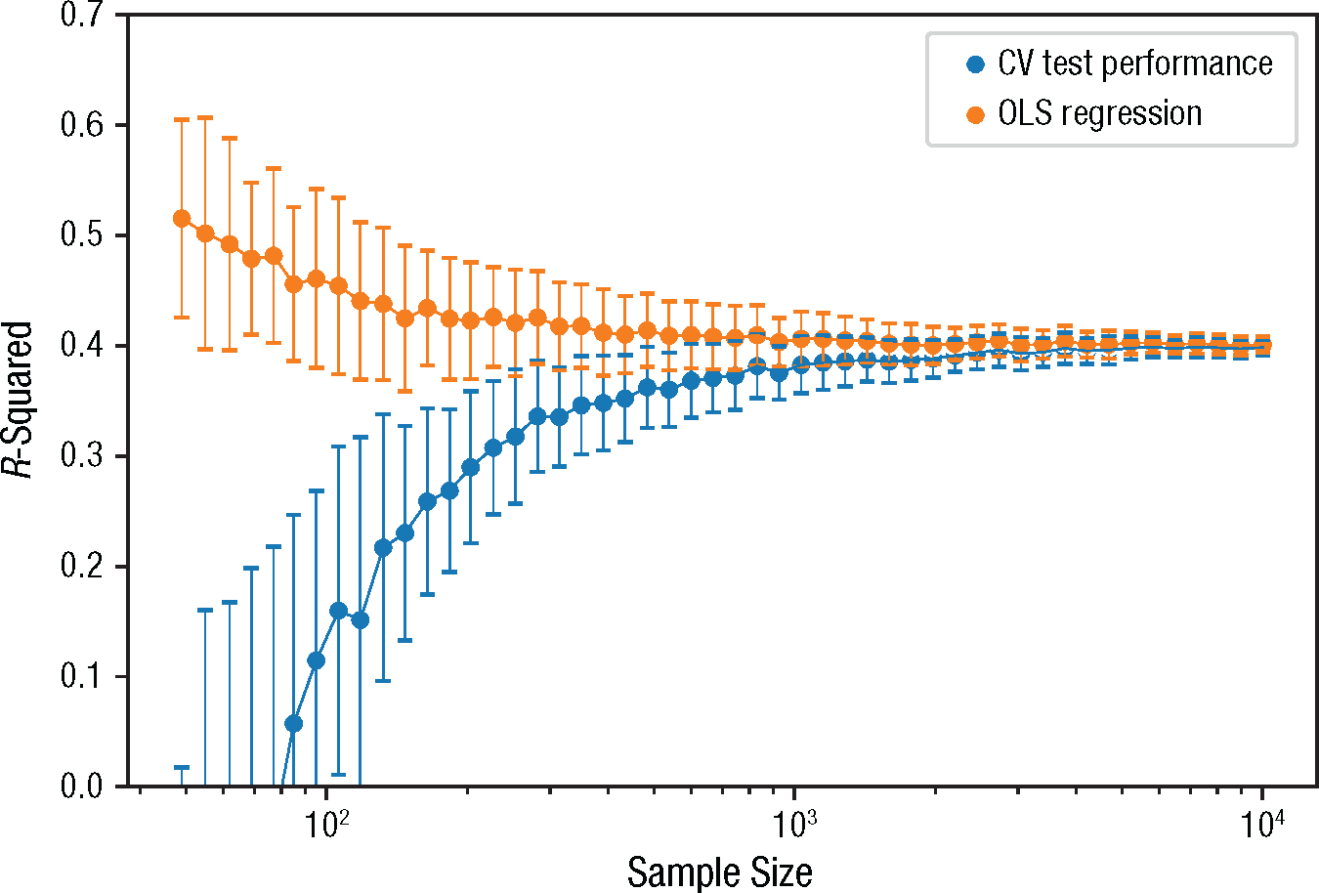
In-sample *R*^2^ from ordinary least squares (OLS) regression and average test *R*^2^ in a 10-fold cross-validated regression on simulated data. Data are drawn from an 11-dimensional multivariate normal distribution (10 used as predictors, one as outcome). All dimensions have zero mean and unit variance. Predictors are orthogonal, and each correlates .2 with the outcome variable. We systematically varied the number of samples drawn (*x*-axis) and repeated sampling and model fitting 100 times per sample size. Error bars represent standard deviation over repeated iterations. For small sample sizes, in-sample performance from OLS regression returns an overly optimistic estimate of the predictive accuracy and generalizability of the model (due to overfitting), whereas out-of-sample cross-validation accuracy returns a more accurate picture of its actual predictive performance. In addition to mitigating overfitting, cross-validation procedures offer the additional advantages of being relatively straightforward to implement and “model agnostic”; they can be applied to virtually any model-fitting framework, which enables direct comparison of any set of models.

Second, the quantitative metrics used to evaluate model performance also need to mean what researchers think they mean and should be appropriately tailored to the intended use case or cases. As discussed earlier, one way in which evaluation metrics in psychology commonly fail to meet this criterion is by relying on in-sample rather than out-of-sample performance estimates. For example, *R*^2^—probably the most widely used evaluation metric in psychology—is typically reported for the very same sample used to fit the model. This approach fails to distinguish signal from noise and will generally produce inflated estimates of a model’s true capabilities (Hofman et al., 2017; Mahmoodi et al., 2017; Shmueli, 2010; Yarkoni & Westfall, 2017). Using cross-validation provides a simple but effective guard against these issues (see Fig. 3).

A related problem is the selection of a metric ill-suited to its intended downstream applications. For example, *R*^2^ is a relative measure of fit (i.e., its interpretation depends on the total variance within a specific sample), and it provides no explicit information about the overall accuracy of individual predictions—in contrast to absolute metrics such as mean squared error or mean absolute error, which allow interpretation of the error associated with individual predictions. In many applications in which psychologists routinely use relative metrics (e.g., quantifying variance explained in psychopathology symptoms), absolute metrics would arguably be more appropriate and more informative with respect to the actual utility of the model for applied predictive purposes.

Ultimately, what counts as a “good” metric will vary from task to task. Metric choice should be optimized for characteristics of the target phenomenon and informed by practical considerations regarding the cost of different types of errors (e.g., low classification specificity and sensitivity can have disproportional practical significance in diagnostic contexts). As for most points discussed in the present section, individual initiatives (e.g., research groups or consortia releasing benchmark data sets, baseline models, and a set of proposed metrics) are likely to play a major role in promoting benchmark development and use on a larger scale and in fostering discussion around the topic.

### Clear practical implications

A large proportion of research in psychology only indirectly affords a direct translation into prediction/explanation of real-world phenomena. Ideally, benchmarks should help counter this tendency by devising predictive tasks grounded in real-world challenges and with clear practical implications. In fact, a large part of what psychologists do already bears a direct relation with applied outcomes (e.g., individuals’ well-being, educational attainment, or clinical profiles). What is required is for researchers to place more emphasis on trying to *predict* and *improve* them. Such a paradigm shift would be desirable for several reasons. First, it would facilitate consensus evaluation of the merits of a theory or model because predictions or key results will often be rendered in intrinsically meaningful quantities (e.g., overt ability to discriminate patients from control participants rather than goodness of fit of a particular latent variable model). Second, models that demonstrably perform well on relatively naturalistic tasks are more likely to successfully generalize to real-world use because there will typically be fewer points of potential failure between model evaluation and model application. Third, the availability of models with clear real-world implications is likely to attract increased interest in and funding for psychology research.

Naturally, the extent to which practical applicability can or should be considered central to benchmark tasks will vary widely across subfields and research questions. Industrial and organizational psychology is a good example of a field in which prediction of practical outcomes (e.g., on-the-job performance) based on variables that can be intervened on (e.g., hiring job candidates with certain characteristics) is a foundational goal. Large-scale studies have been conducted targeting applied challenges such as predicting team effectiveness from wearable sensor data capturing team interactions and communication patterns (Kozlowski & Chao, 2018) or using text mining to identify issues with customer satisfaction (for an overview of big data studies in I/O psychology, see Guzzo et al., 2015). In this field, developing benchmarks with an applied angle would merely require coordination and scaling of existing resources.

Predicting real-life outcomes is also an important goal in educational psychology (in which understanding the factors that affect educational achievement can directly inform teaching strategies and education policies), in political psychology (in which large-scale surveys aimed at informing prediction of voting behavior are periodically conducted and many efforts are devoted to developing text models detecting toxic or misleading political contents), and in several domains of clinical research (in which predictive models are used for purposes ranging from causal modeling of psychiatric diagnoses to predicting patients’ risk of being subjected to mechanical restraint; see Danielsen et al., 2019). Other clinical fields have fewer readily available data sets but also have high potential. For example, brain stimulation data sets could be used to develop models yielding individualized predictions of the boundaries of specific brain circuits (e.g., language areas) and have direct applications for presurgical mapping based on functional MRI (fMRI; Benjamin et al., 2017; Bookheimer, 2007). In this and other cases, efforts to develop relevant resources could significantly promote progress in solving challenges of high practical significance.

### Data sharing infrastructure and open-source software

Large data sets, solid metrics, and an applied focus are important foundations of good benchmarks, but their utility is often limited by the absence of suitable infrastructure and tools. Ideally, good benchmarks should be released alongside resources that enable users to readily access and manipulate benchmark data sets; to train, test, and deploy models; and to publicly share validation results. The availability of high-quality and domain-general infrastructure for storage, sharing, and querying of large data sets is currently limited in psychology, although the situation is rapidly changing for the better. For data storage and sharing, OSF (https://osf.io) is the most widely used platform. OSF provides storage and sharing capacity for a wide variety of data and resources (not limited to psychology). However, it comes with no data-sharing standards, and resources to programmatically access OSF material are still limited. The neighboring domain of neuroscience is an excellent source of inspiration on how to overcome these challenges. An integrated suite of tools that includes a platform for data sharing (OpenNeuro; see Gorgolewski et al., 2017), data-sharing standard (BIDS; see Gorgolewski et al., 2016), and tools for programmatic access of BIDS data such as PyBIDS (Yarkoni et al., 2019) has been developed over the last few years. There are promising signs that other areas of psychology are moving to adopt a similar model—for example, there is an incipient effort to develop a lightweight, BIDS-like specification for representing psychological data set (Psych-DS; https://psych-ds.github.io/).

Complementary to improvements on the data-sharing side, high-level tools should be developed that enable easy access to benchmark data sets. A potential source of inspiration is the nlp library developed by HuggingFace, which enables easy programmatic access to popular data sets for natural language processing. Furthermore, software facilitating training and deployment of models on reference data sets would be highly beneficial. Examples of similar tools (coming, again, from natural language processing) are libraries such as HuggingFace’s transformers, or broader scope ones such as spaCy and AllenNLP (Gardner et al., 2018). These libraries offer functional routes and pipelines to easily train, fine-tune, evaluate, and deploy models, which makes natural language processing tools accessible beyond the narrow community of specialists. Availability of similar packages for psychology would also make data sets and models readily available as a “service” for and beyond the research community and encourage their deployment in practical (e.g., societal or technological) applications. Such tools could also be conveniently paired with packages for model diagnostics, such as checking for basic validity criteria like falsifiability or identifiability (I. J. Myung & Pitt, 2002; Oberauer et al., 2018) or performing post hoc tests on model predictions to identify biases.

As the example of the BIDS neuroimaging ecosystem attests, organizing recurring events (workshops, conferences, hackathons) and setting up consortia dedicated to defining the structure of these tools can be an effective way to promote their development. Openness, integration, and community-based development should be core principles of this endeavor.

### Well-aligned incentives

Most of the benefits of the benchmarking practices we have described apply at the level of entire research communities. But to attain widespread adoption, such practices also need to be incentivized at the individual level. Here, machine learning again offers a source of inspiration. In machine learning, it is common practice to release benchmarks in combination with competitions that encourage the development of systems that can advance the state of the art on the target task. This competition-oriented culture is perhaps inherited from artificial intelligence, whose development has been profoundly shaped by the goal of outperforming humans on challenging intelligence tasks (notable examples are the chess match between Garry Kasparov and IBM’s Deep Blue chess computer or the Go match between Lee Sedol and DeepMind’s AlphaGo model), and it has been fostered by milestone events such as the 2006 Netflix Prize Competition (Bennett & Lanning, 2007) and the already mentioned ILSVRC.

Over the years, predictive challenges have become increasingly popular beyond machine learning. This competition-oriented and community-based model development framework is today prominently embodied by Kaggle (https://www.kaggle.com/competitions), a platform hosting and promoting data science challenges, which simultaneously advances funders’ interests, encourages technical progress via community-driven efforts (Garcia Martinez & Walton, 2014), and provides an important training ground for both budding and expert data scientists. Contests and competitions are also gaining momentum in cognitive neuroscience, a field that places increasing emphasis on data-sharing resources and tools for reproducible data analysis or reanalysis. A notable example is the ongoing TReNDS Neuroimaging Kaggle competition (https://www.kaggle.com/c/trends-assessment-prediction), in which the goal is to develop models that robustly predict multiple normative assessments from multimodal MRI features.

Promoting contests and competitions is one of the measures that could prove effective in encouraging researchers to adopt benchmarking practices on a large scale. As we discuss in later sections, however, excessive emphasis on leaderboards also has serious downsides (e.g., the tendency to favor high-resource groups and computationally intensive solutions, which comes at high environmental costs). Competitions should therefore be accompanied by a set of complementary, systemic incentives. One important domain is that of publication standards and practices. Clear and detailed model descriptions and (wherever possible) reporting of predictive performance on benchmark tasks (alongside metrics of model complexity and resource requirements) should be demanded when reviewing articles making quantitative claims. More publication formats tailored to data set, model, or metrics development may be introduced to incentivize scientists to invest more time and resources in this type of work (some publication outlets relevant to psychology already offer this option).

### Adaptiveness

Good benchmarks dynamically adapt over time to the goals and developments of a field: They should evolve and improve, with new benchmarks being introduced as the field evolves and old benchmarks occasionally becoming obsolete when performance plateaus or when their intrinsic limitations become apparent. Machine learning offers an interesting example of the latter scenario—one that illustrates how a heavy emphasis on benchmarking and consensus metrics can often encourage methodological debate rather than silencing it.

The example comes from computer vision, in which it has been noted that the majority of models that achieve near-perfect performance on ImageNet classification tend to perform very poorly in real-world applications (Barbu et al., 2019). Low reliability of benchmark metrics in reflecting models’ predictive performance in real-world tasks has been attributed to limitations intrinsic to the ImageNet data set: The images display only objects as seen from a limited set of viewpoints and on a limited set of background types. Systems trained on ImageNet may thus learn to identify objects according to data-set-specific features of images that are poorly correlated with the corresponding object classes in the real world, which leads to poor generalization in naturalistic scenarios. These considerations have led to the development of a new benchmark, ObjectNet (Barbu et al., 2019), in which features of the visual context such as viewpoint and background are systematically controlled for. Performance of traditional computer-vision architectures on ObjectNet drops dramatically compared with ImageNet, which has readjusted beliefs on the capabilities of state-of-the-art models, and encourages the development of new models that can overcome these weaknesses.

Note that benchmarks should be continually evaluated and revised not only for performance-related reasons but also for ethical ones. Benchmarks are introduced in specific historical and social contexts, and they may not be immune to severe biases. A critical reevaluation of machine-learning benchmarks over the last years has revealed the presence of racial and gender biases in data sets and models, which has boosted interest in fair artificial intelligence (Zou & Schiebinger, 2018) and highlighted the need for a radical rethinking of research practices and the structure of research communities (Kalluri, 2020). This, too, is an example of how dynamicity should be considered a feature rather than a bug. Bias issues in machine learning are far from solved. Openness to rethinking reference data sets, models, and evaluation paradigms enables the community to iteratively evolve increasingly more ethical scientific practices.

Benchmarks that satisfy all the criteria outlined in this section can dramatically improve the current state of model evaluation in psychology, promote cumulative scientific progress, and help overcome other major liabilities in research practices. Such benchmarks would not only provide objective and reliable criteria for “model-agnostic” (Ribeiro et al., 2016a, 2016b) assessment of model performance—that is, evaluation criteria independent of the specific characteristics of and structural similarity between models—but also encourage researchers to adopt more transparent research practices by formalizing their models and making their theoretical assumptions and operationalizations of target constructs explicit. However, the degree to which all these conditions can be fulfilled varies widely across fields.

## Existing Work and Feasibility Across Fields

We have outlined some core principles that would facilitate the development of benchmarks that fit the overall characteristics and goals of psychology. Psychology, however, is a complex and multifaceted domain: Its subfields vary in their methodological profile, in the importance attributed to prediction of real-world outcomes, and in the extent to which they are sensitive to the need for common metrics. As a result, some fields have already come close to developing evaluation benchmarks, whereas others still struggle to introduce consensus metrics and big data into standard workflows. In what follows, we present examples of fields in which practices favorable to the introduction of benchmarks are already established and point the way to further developments. We then provide examples of fields in which such practices are less common and discuss how existing resources could be leveraged to promote them.

### Qualitative benchmarks in working memory research

One of the fields that has come closest to introducing evaluation benchmarks is that of short-term and working memory research, in which development of benchmarks for model and theory evaluation has been explicitly advocated and undertaken in work by Oberauer et al. (2018). The authors ground their attempt to develop benchmarks for short-term and working memory research in the observation that the number of novel empirical findings in psychology has been growing so dramatically over the years that no psychological theory can realistically account for all of them. When testing their theories, researchers thus inevitably select a preferred subset of facts or even manufacture novel ones using new experimental paradigms. This results in most psychological theories having nonoverlapping empirical scope, which makes objective comparison difficult or impossible. In the authors’ view, introducing benchmarks would allow researchers to focus theory-development efforts on a set of substantive findings that any valid theory should account for and to objectively compare models on their ability to do so.

The project undertaken by Oberauer and colleagues (2018) provides an excellent model for how to practically develop a set of consensus evaluation tasks and metrics collaboratively and transparently. Benchmark development was carried out by a consortium that gathered a team of scientists from a variety of research institutions and theoretical backgrounds, and their collaboration unfolded over a series of seminars and workshops. Updates on the project were progressively reported on the project’s webpage (https://wmbenchmarks.wordpress.com), from which a public forum also gathered input from the broad research community. The set of benchmarks developed over the course of the project is now publicly available on GitHub (https://github.com/oberauer/BenchmarksWM).

Our proposal aligns with this outstanding work on the motivation and need for benchmarks; however, there are a few differences in the proposed approach. First, in Oberauer and colleagues’ (2018) proposal, benchmarks are designed to evaluate models on the extent to which their predictions globally capture solid and replicable statistical effects. We argue that models should ideally also be evaluated on their ability to quantitatively predict individual data points. Furthermore, in our view, benchmarks should privilege predictive challenges that focus on complex, naturalistic data to favor models whose predictions generalize to real-world problems, rather than aiming at merely replicating well-established findings from controlled experimental settings. We recognize, of course, that the feasibility of this goal varies largely across fields.

### Genome-wide association studies and polygenic scores: the case of statistical genetics

A second field that offers useful parallels for psychology is statistical genetics. Research in statistical genetics focuses on clarifying to what extent and how phenotypic variation along complex behavioral traits (be it binary traits, such as diagnoses, or quantitative traits, such as personality dimensions) can be attributed to heritable genetic influences (Fisher, 1919; Wright, 1920) and various environment factors. Although we are not aware of efforts to explicitly introduce common benchmarks to this literature, many of the features we have advocated for are already embedded in standard workflows.

Multisite data collection and data sharing have long been common in statistical genetics (Haworth et al., 2013; Hur & Craig, 2013). In earlier stages of the discipline, in which techniques for direct, large-scale inspection of the genome were not yet available, heritability had to be estimated indirectly through twin and family studies (Plomin et al., 2016), which require gathering data from individuals in rather rare groups and configurations. Novel data-collection and analysis techniques, however, have made large data sets and public data sharing even more necessary. One key development has been the introduction of genome-wide association studies (GWASs; see Visscher et al., 2012), which can support identification of specific genome-phenotype associations. Because complex traits are highly polygenic (Chabris et al., 2015), adequately powered GWASs require sample sizes on the order of at least tens of thousands (Dudbridge, 2013). This has provided even further incentives for the development of large-scale collaborations (e.g., the Wellcome Trust Case Control Consortium, https://www.wtccc.org.uk/) and infrastructure for effective data sharing (e.g., GWAS central, https://www.gwascentral.org/).

Findings from this line of work have dramatically reshaped beliefs about the complexity of the genetic mechanisms underlying most phenotypes and have led researchers to reconsider many findings from candidate gene studies now deemed spurious (Border et al., 2019; Chabris et al., 2015; Duncan & Keller, 2011). Note that GWASs have also facilitated the emergence of consensus on target metrics and promoted heavier emphasis on prediction (de Los Campos et al., 2013, 2015; Makowsky et al., 2011). Polygenic (risk) scores (i.e., linear combinations of effect sizes for each single nucleotide polymorphism) are now commonly used to predict an individual’s genetic liability to a disease or a trait. Consensus on polygenic scores as a standard metric and the availability of increasingly large data sets and refinement of analysis techniques (e.g., the introduction of genetic complex-trait analysis; see Yang et al., 2010, 2011) have enabled researchers to make significant progress in prediction of behavioral traits over the last decade. One notable example is educational achievement. Whereas early GWAS-based models explained only around 2% of the variance (Rietveld et al., 2013), current models explain as much as 20% (Allegrini et al., 2019; Lee et al., 2018; Selzam et al., 2018; von Stumm et al., 2020). Similar improvements in levels of predictive performance can be observed for other complex phenotypes, such as personality (Power & Pluess, 2015; Vinkhuyzen et al., 2012) and psychiatric traits (Schizophrenia Working Group of the Psychiatric Genomics Consortium, 2014; Visscher et al., 2012; Wray et al., 2014).

### Lexical norm data sets and megastudies in psycholinguistics

Similar to behavioral genetics, psycholinguistics has witnessed a number of developments that have made it amenable to the introduction of evaluation benchmarks. The long-standing popularity of lexical decision paradigms has guaranteed a certain degree of methodological consensus, especially in research on lexical processing and semantics, and the use of large data sets has become common practice.

A number of large-scale lexical norms data sets have been developed and publicly shared (especially for English and Dutch) over the last decades. The first significant effort in this direction was the MRC Psycholinguistic database (Coltheart, 1981), which gathered semantic, syntactic, and lexical information for about 98,538 words, albeit many of these norms are now obsolete. More recent megastudies (Kessler et al., 2002; Spieler & Balota, 1997; Treiman et al., 1995; for a comprehensive list, see http://crr.ugent.be/programs-data/megastudy-data-available) have indexed increasingly large portions of the lexicon on wider sets of variables (Brysbaert et al., 2014; Brysbaert & New, 2009; Chateau & Jared, 2003; Kuperman et al., 2012; Tucker et al., 2019; Warriner et al., 2013). Among the most notable examples is the English Lexicon Project data set (Balota et al., 2007), which includes a wide range of psycholinguistic norms and reaction time data gathered from a large participant sample for around 40,000 words and around 40,000 non-words. More recently, data sets explicitly targeting psychologically and neurologically grounded features (Binder et al., 2016; Lynott et al., 2020) and word association norms (De Deyne et al., 2016, 2019) have been developed and made publicly available.

Development of large-scale data sets has not only speeded up the transition to big data in psycholinguistics but also has contributed to a moving away from significance testing in favor of increased focus on model performance—although mostly still based on goodness-of-fit metrics. Furthermore, the use of large-scale reference data sets has enabled convergence on a set of consensus predictive tasks of common interest and on the operationalization of relevant variables, which paves the way for the introduction of common evaluation benchmarks.

### Other potential applications

Beyond these examples, there are many other domains in which existing resources could be adapted into useful benchmarks or in which entirely new data sets could be acquired with this goal in mind. As a general heuristic, we expect that an emphasis on benchmarking will prove most productive in fields that have an applied focus, a robust methodological apparatus (solid constructs and consistent operationalizations), and/or agreement on core empirical problems and target metrics. However, there is no predefined set of criteria defining which fields are likely (or eligible) to develop into prominently prediction-based and benchmark-based fields. Ultimately, successful introduction of common evaluation benchmarks will depend on the community’s willingness to engage in constructive methodological rethinking and on individual efforts to pioneer benchmark development. Even in fields that do not necessarily check the above boxes, thinking about potentially useful benchmarks is possible, and it can draw much needed attention to the practical implications (or lack thereof) of existing research programs. Below, we provide a few examples drawn from diverse areas of psychology that vary considerably in feasibility and ambitiousness:

- In educational psychology, prediction of student outcomes from individual features (e.g., demographics, proxies for personality/cognitive profiles) and features of the learning portfolio is an example of a task with immediate practical applicability. High-performing models could, in fact, not only be used for theoretical purposes, that is, to gain a better understanding of how individual profiles and teaching strategies interact in shaping successful learning experiences, but also deployed as tools for education professionals to tailor teaching strategies to target audiences or for individuals to optimize their educational choices (e.g., choice of higher education programs) on the basis of their own profile (for an overview of existing work using machine learning for precision education, see Luan & Tsai, 2021). Efforts to develop similar tools driven by the research communities and conducted in partnership with institutions would be highly beneficial to the public and provide a transparent, ethical, nonprofit framework that could help improve public education systems. A number of useful resources already exist that could be gathered into large data sets to train and evaluate relevant models. A number of international surveys are periodically conducted that target student outcomes at different ages and in a variety of domains and that also gather information about student background, demographics, attitudes, home environment, and school characteristics. Relevant resources include Program for International Student Assessment reports as well as data from the Progress in International Reading Literacy Study, Trends in International Mathematics and Science Study, and Program for International Assessment of Adult Competencies surveys. But there is also considerable opportunity for development of valuable new data sets. Massive open online courses, for example, are potentially rich data sources particularly suited to the purpose of benchmark development both by virtue of their scale and the advantages yielded by their natively digital format. Course metadata (e.g., duration and type of video lectures, degree of interaction between students, number of practical exercises, and amount of group work involved), automated annotations of the teaching material (e.g., quantification of linguistic styles and prosodic traits in videos), student feedback, and background information on learners’ profiles are some examples of features that could be extracted and used to develop models targeting prediction of student outcomes.
- Traditionally, personality psychology has almost exclusively focused on profiling personality by using questionnaires and self-reports. Yet personality assessments based on traditional approaches have low predictive validity when tested on real-world outcomes, especially when aggregated traits are used as features rather than individual items (see Mõttus et al., 2017; Revelle et al., 2021; Saucier et al., 2020; Wiernik et al., 2020). Furthermore, self-reports are affected by biases (Kreitchmann et al., 2019; Müller & Moshagen, 2019), and standard questionnaires are extremely time-consuming, often consisting of hundreds of items, which intrinsically limits scalability. The availability of social media data is a potential game changer for data-driven approaches to personality modeling given that personality traits are likely expressed in online behavioral patterns. Building large data sets pairing social media data (e.g., Reddit submissions, https://reddit.com) with known personality scores and/or indices of real-world outcomes (shared, of course, with consent and safe data-protection protocols) could pave the way to the development of innovative (and potentially predictively powerful) approaches to personality modeling. Some attempts in this direction have already been made (e.g., predicting personality from Facebook likes or word use, see R. M. Brown et al., 2018; Park et al., 2015; or from musical preferences, see Nave et al., 2018). But in general, because of the scarce availability of suitable open-access data sets, these approaches have been mostly beyond reach for the academic community and remain a prerogative of corporate research.
- A large proportion of clinical research has real-world prediction as its immediate goal and aims at early detection, prevention, monitoring, or treatment of clinical conditions. Extensive efforts are being made in clinical psychology to standardize measures and screening procedures administered to participants at intake of various studies as well as outcome measures administered to participants at fixed follow-up intervals. These developments are highly favorable to the creation of benchmarks (potentially paired with competitive challenges) enabling evaluation of models predicting, for example, changes in measures of global function, attrition, or other clinically useful targets using intake measures (either collapsing across all treatments or separately for specific interventions).
- Prediction research in political and social psychology can inform policymaking or even guide intervention aimed at countering the emergence of toxic societal dynamics. A large amount of work has been devoted, for example, to modeling the dynamics of polarization. Models able to predict the emergence of extreme-polarization scenarios or even explicit violent outbursts of social conflict would be a highly valuable tool for policymakers and other agents involved in shaping public discourse. Once again, social media are highly relevant data sources. Data from social networks allow modeling of semantic and pragmatic aspects of public discourse and the dynamics of social interactions (e.g., the formation of echo chambers), both likely linked to levels of social polarization. Pairing these data with explicit indices of social polarization (e.g., periodic surveys) or conflict outbursts (e.g., relevant news events) would provide researchers with valuable resources to conduct predictive work on the matter. In the domain of political psychology, many other interesting challenges could be tackled by harvesting and adapting existing resources. Political prediction markets are another promising example. Prediction markets data could be leveraged not only to inform the understanding of what drives changes in political affiliation and beliefs but also to develop and evaluate models able to forecast the outcome of key political events (Wijesinghe & Rodrigues, 2012).
- In social psychology, efforts have been made to develop large data sets that enable fine-grained modeling of social network structures alongside individuals’ psychological traits and behavior. One example is the Copenhagen Networks Study, which involved tracking the location and digital interactions of a large cohort of students over an extended period of time and profiling their personality and cognitive traits (see Sapiezynski et al., 2019). Similar longitudinal data sets could be leveraged to develop growth models predicting, for example, student dropout, performance, or likelihood to seek mental health help.

## Common Pitfalls and Concerns

Benchmarks have the potential to revolutionize model evaluation practices in psychology in favor of higher reliability, objectivity, and practical utility. They are not, however, exempt from potential drawbacks. Furthermore, the strong emphasis placed on predictive validity is likely to raise (more or less well founded) concerns in the research community. We now review several caveats, potential pitfalls, and common objections that deserve special attention and provide suggestions on strategies to sidestep them wherever possible.

### Generalization is hard

Some metrics are better than others in yielding trustworthy information on model performance. We have already highlighted how metrics that explicitly account for the risk of overfitting and value out-of-sample predictive accuracy tend to be more reliable than commonly used goodness-of-fit metrics. Still, no metric is perfect. The extent to which performance estimates from predictive metrics generalize to unseen data depends on characteristics of the data set, even for robust metrics such as cross-validation criteria. Small and/or nonrepresentative samples often yield unreliable estimates. High predictive accuracy on data generated using a single experimental paradigm, for instance, may be an artifact of methodology-specific biases in the sample (e.g., systematic measurement error), and those accuracy levels may not generalize to samples collected using different methodologies. Metrics such as the generalization criterion (Busemeyer & Wang, 2000) attempt to address this issue by imposing the constraint that validation sets should come from experimental designs different from those used in training. This is, however, only one of the many ways in which samples can fall short of being fully representative of the phenomenon or population of interest. Nonrepresentative participant samples or stimulus sets may also affect generalizability to novel data.

Note that generalization issues cannot always be fully solved through adoption of good practices such as cross-validation and out-of-sample evaluation. Concerns about result replicability and generalizability have also been raised for machine-learning models (Emmery et al., 2019; National Academies of Sciences Engineering and Medicine, 2019; Vijayakumar & Cheung, 2019). As we discuss in the next sections, careful design of large, representative samples; adoption of multibenchmark model evaluation; and dynamic updating of reference benchmarks (as in the ImageNet case) are further measures that could help limit (although not fully eradicate) the risk of generalization flaws in predictive contexts.

### Goodhart’s law: when a measure becomes a target

Specific benchmarks can prove extremely useful in certain stages of development of a discipline. But their limitations tend to emerge as soon as they become the primary or only criterion to evaluate model performance. As Goodhart’s law states, “when a measure becomes a target, it ceases to be a good measure.” As the ImageNet versus ObjectNet example discussed earlier illustrates, when optimizing for a specific task becomes the exclusive focus of model engineering efforts, researchers may lose sight of the fundamental goal of generating good predictions on other (e.g., real-world) instances of the task at stake. The development of good benchmarks should consequently always be viewed as an ongoing process in which tasks and metrics are continuously evaluated, reevaluated, and updated.

One way to mitigate Goodhart’s law is to develop multidimensional benchmarks (i.e., batteries of tasks on which models are jointly evaluated). The concept of *transfer learning* in machine learning captures a similar principle—models trained to optimize performance on one set of tasks are evaluated according to their performance on multiple tasks (potentially including entirely new ones) without updating (or fine-tuning) parameters separately for each task (for an example and an overview of transfer learning in natural language processing, see McCann et al., 2018; Ruder et al., 2019).

Once again, note that these measures cannot protect from all potential side effects involved in placing *exclusive* emphasis on predictive accuracy. Recent literature has highlighted a number of scientific and ethical shortcomings associated with common machine-learning practices, especially with respect to recent developments in natural language processing (for a comprehensive overview, see Bender et al., 2021; Bender & Koller, 2020). These may not apply to psychology in the short term, but it is important to factor them in when designing benchmarks (e.g., by developing evaluation tools attuned to ethical concerns) and learn critically from the experience of machine learning rather than blindly imitating its mistakes.

### Beyond predictive accuracy: validity, parsimony, and the ethical cost

High predictive accuracy is, of course, not the only criterion models should be evaluated on in psychology, and our advocacy of benchmarking is not meant to discourage researchers from taking other criteria into account when designing or evaluating models. Indeed, good predictive performance can sometimes be achieved by models that fail basic validity tests (J. I. Myung & Pitt, 2018).

Criteria such as simplicity and parsimony are also often important considerations in the choice of model. There are several reasons for this. A sophisticated algorithm that performs well but is too complicated for a clinician to effectively apply in a real-world setting may be less useful than a “fast-and-frugal” heuristic that performs more poorly under optimal conditions (Gigerenzer et al., 1999). And in cases in which predictive performance is roughly comparable, simpler models are generally more desirable; fewer parameters reduce the risk of overfitting and the amount of compute required for training and deploying. Conversely, large models can easily become prohibitive in terms of computing resources; they may come with large environmental costs (Strubell et al., 2019), which disproportionately affect populations that are not from Western, educated, industrialized, rich, and democratic societies and pose ethical dilemmas; and their complexity may not be entirely justified in terms of performance gains. Some state-of-the-art transformer language models, for example, have proven to be over-parameterized (Clark et al., 2019; Kovaleva et al., 2019): They contain many idle architectural units, and their performance levels are matched by smaller models trained using parsimonious schemes such as model distillation (e.g., Sanh et al., n.d.). It is thus important to incentivize researchers to strive for parsimonious solutions whenever possible—for example, by introducing the custom of quantifying and reporting complexity and resource requirements alongside any mention of predictive performance (several proposals in this direction have been advanced in machine learning; e.g., see Henderson et al., 2020; Rogers, 2019).

At the same time, increased focus on benchmarking could potentially help discourage the somewhat common tendency to dismiss complex models out of hand merely because of their complexity or because of more subjective considerations. Qualitative criteria such as model plausibility, explanatory adequacy, or faithfulness, which reflect the extent to which models are grounded in existing literature or theoretical considerations, are often also advocated as central to model evaluation (I. J. Myung & Pitt, 2002; J. I. Myung & Pitt, 2018). However, the question of what role these criteria should play relative to clever model engineering is an open one. We lean toward a solution in which qualitative constraints should, in general, not play a role in benchmark-based evaluation (e.g., model scores should not depend on a panel of judges’ subjective assessment of theoretical elegance).

This does not mean that considerations such as model complexity, computational efficiency, and so on cannot be explicitly factored into a model’s score on a benchmark, only that the model’s score should be a deterministic function of clearly stated metrics and should not depend on irreproducible components. Our position should also not be taken to imply that subjective considerations are unimportant in model evaluation; indeed, we think that a large element of subjectivity is probably unavoidable in psychology given the complexity and underdetermined nature of most psychological phenomena. But at the very least, researchers should strive to make it clear where objective metrics end and subjective valuations begin.

### Interpretability

As we argued above, to achieve maximal reliability and utility, benchmark-based evaluation should rely on metrics that adequately capture the predictive validity (and, therefore, the generalizability) of a model. However, one particularly common objection to a push for greater emphasis on predictive accuracy is that models that yield higher predictive accuracy are intrinsically less interpretable than simpler (e.g., linear) ones and thus fail to contribute to understanding the empirical phenomenon of interest.

It is true that in many cases, the models yielding best performance are the more complex ones because more parameters reduce prediction bias and offer the flexibility needed to capture complex patterns in the data. It is also true that parameters from complex models (e.g., weights in deep-learning models) often lack a straightforward direct interpretation. However, the argument that higher complexity corresponds to lower interpretability—and for that matter, that interpretability is intrinsically desirable—builds on a number of questionable assumptions. Debunking these fallacies is especially important because these arguments are among the reasons why complex models (e.g., deep-learning models) are often “discarded for scientific purposes such as theory building and testing [omit understanding]” (Shmueli, 2010, p. 291).

One of the assumptions behind the interpretability counterargument is that simpler (linear) models are more straightforwardly interpretable than complex (e.g., non-linear) ones. This statement is far from uncontroversial. Direct interpretability of individual parameter estimates in linear models is, in fact, highly dependent on overall properties of the model (i.e., covariates, degree of collinearity, interactions), and although researchers in the explanatory tradition often tend to forget it, parameter estimates are always conditional on the model itself.

This point is nicely illustrated by recent neuroimaging and psychology studies (Botvinik-Nezer et al., 2020; Silberzahn et al., 2018) in which independent research teams were asked to test a given set of hypotheses on the same data set. Analytical approaches adopted by the teams can vary along a number of parameters (e.g., choice of covariates, statistical tests, coding procedures) and result in quantitatively different feature coefficients and thus, potentially, in qualitatively different interpretations of the effect of such features. This variability in estimates cannot always be explained away by disparities in performance. Even models achieving comparable predictive accuracy can diverge widely in the importance attributed to individual features (Churchill et al., 2014; Schmah et al., 2010) regardless of the similarity between the models’ structure or analytical form.

The flip side of this assumption is the idea that complex models are intrinsically uninterpretable, or, to put it more mildly, that they are much less interpretable than the cognitive processes they are meant to model. This common misconception likely stems from a certain ambiguity in the way interpretability is defined is the literature, but it does not ultimately stand up to scrutiny under any plausible definition (Lipton, 2018). If interpretability is defined in terms of transparency, that is, as the possibility to directly observe the sequence of algorithmic steps through which a decision/output has been produced, human cognitive processes are obviously no more interpretable than complex models (and probably much less so). If, on the other hand, interpretability is defined as the possibility to provide a post hoc, compact, natural language explanation of why a certain output was produced in response to a certain input, then humans and complex artificial models can, in principle, be equally interpretable. Saliency maps (Simonyan et al., 2013), behavioral testing (Ribeiro et al., 2020), probing methods (Bolukbasi et al., 2016; Bordia & Bowman, 2019; Gardner et al., 2020; Kim et al., 2019; Linzen & Baroni, 2020), game-theoretic feature attribution methods (Lundberg & Lee, 2017), and adversarial attacks (Goodfellow, Pouget-Abadie, et al., 2014; Goodfellow, Shlens, & Szegedy, 2014) are among the techniques that can be used to make the behavior of machine-learning models more transparent (for a more comprehensive overview of interpretability research in machine learning, see Molnar, 2020), which can, in turn, inform theoretical accounts of target phenomena.

As these examples show, emphasis on predictive accuracy and, consequently, increased reliance on complex models in psychology need not result in a loss of interpretability. Likewise, it does not imply relinquishing ambitions of scientific understanding: Even highly complex models can be probed and compared in ways that can inform mechanistic explanations of cognitive phenomena. Once again, machine learning offers some useful examples. A few studies have already shown how systematic comparison of benchmark performance of language models (e.g., Talmor et al., 2019) that differ in the presence/absence and type of self-attention mechanism or in the size of input context (long- vs. short-context models) can be used to corroborate theoretical intuitions on key aspects of human language processing (e.g., the role and time scales of predictive processing and context integration in online language understanding). For instance, in a recent study, Schrimpf et al. (2020) compared the performance of different language model architectures in predicting psycholinguistic and neural language comprehension data and showed that whereas bidirectional attention models perform best on traditional natural language processing tasks, unidirectional language models predict fMRI data best. Recent work has more generally advocated for the development of cognitive benchmarks for language models (Artemova et al., 2020; Hollenstein et al., 2019), which would enable comparative assessment of the cognitive plausibility of natural language processing models but also shed light on key characteristics of the cognitive underpinnings of human linguistic behavior.

The possibility to retain control over the heuristics complex models develop to generate predictions makes sure that stronger emphasis on prediction will not alter psychology’s ability to cumulatively build knowledge whose scope goes beyond practical applicability. Predicting and generating knowledge about phenomena are incompatible goals only if the latter is reduced to the impoverished notion of explanation intended as inference about (causal) relationships between variables achieved through direct interpretation of model estimates (which, as we argued above, is a deeply problematic one). There are several alternative and more fruitful pathways to generating knowledge through predictive modeling, some combining computational research practices with insights from experimental design familiar to psychologists. These “mixed” approaches are far from new to psychology and its neighboring fields; similar frameworks have been widely adopted, for example, in agent-based modeling research.

### What role for domain expertise?

A final concern associated with an increased emphasis on benchmarking is that achieving good predictive performance on many tasks might turn out to require less traditional domain expertise than many researchers currently suppose. That is, it may turn out that the skills and knowledge bases needed to build good applied models in psychology have relatively little overlap with those emphasized in traditional psychological theorizing. This worry is not entirely without basis; it has been frequently observed that the winners of prediction-focused competitions, even when ostensibly focused on narrowly domain-specific problems, disproportionately tend to be teams of machine-learning experts or data scientists with relatively little, and sometimes no, domain expertise (e.g., AlQuraishi, 2019; Bentzien et al., 2013).

We think that such a prospect—although perhaps uncomfortable in some ways—should, if anything, increase the utility of benchmarks in psychology. If it turned out that achieving good performance on major benchmarks in applied fields such as educational psychology, psychopathology, and I/O psychology primarily requires computational rather than substantive expertise, this would constitute a powerful argument for emphasizing those skills to a greater extent in our training programs. To the degree that we believe that the public’s investment in psychological science is ultimately aimed at improving the human condition, one might even argue that we have a collective moral obligation to subject the field to rigorous challenges of this kind—in much the same way that we have advocated for the objective evaluation of individual models.

## Conclusion

Lack of consensus on robust metrics for model evaluation has long hindered progress in psychology. In this article, we argued that introducing benchmarks can help overcome this fundamental issue, and we provided general guidelines and concrete suggestions on how to go about developing metrics that suit the multifaceted profile of the field. Benchmarks inspired by real-world predictive challenges will not only provide psychology with communal evaluation metrics but also motivate the community to redirect efforts toward solving problems with practical implications and high societal relevance.

## Footnotes

1. The code for these analyses is available on GitHub at https://github.com/rbroc/benchmarks_paper.

2. Some readers may question this value, under the belief that *R*^2^ values must have a lower bound of zero. But under the traditional definition of *R*^2^ (i.e., 1 – the ratio of residual to total variance), it is possible to have an arbitrarily large negative *R*^2^. Values below zero imply that the model performs worse than a constant model that simply uses the sample mean as the prediction for all individual observations.
